# Comparison of Portal Vein Doppler Indices and Hepatic Vein Doppler Waveform in Patients with Nonalcoholic Fatty Liver Disease with Healthy Control

**DOI:** 10.5812/kowsar.1735143X.729

**Published:** 2011-09-01

**Authors:** Ehsan Solhjoo, Fariborz Mansour-Ghanaei, Roghaeyh Moulaei-Langorudi, Farahnaz Joukar

**Affiliations:** 1 Radiology Department, Guilan University of Medical Sciences (GUMS), Rasht, IR Iran; 2 Gastrointestinal and Liver Diseases Research Center (GLDRC), Guilan University of Medical Sciences (GUMS), Rasht, IR Iran

**Keywords:** Hepatitis, Fatty liver, Ultrasonography, Alcohol

## Abstract

**Background:**

The purpose of the present study is to investigate the association of nonalcoholic fatty liver disease (NAFLD) with the doppler waveform pattern of hepatic veins and portal vein doppler indices.

**Objectives:**

This assay may be useful in evaluating the natural course of NAFLD and monitor treatment efficacy on follow-up.

**Patients and Methods:**

This case control study was performed in 31 patients with NAFLD and 31 normal healthy adults who served as the control group. The patients presented with elevated liver enzymes levels (ALT/AST) and hyperechogenic livers in the B-mode ultrasonography examination. Eleven patients had a liver biopsy. After an 8-hour fast,B-mode and duplex doppler ultrasonography were performed, and the waveform patterns of the right hepatic vein, portal vein diameter, grade of fatty liver, portal vein pulsatility index (VPI), and mean flow velocity (MFV) were measured.

**Results:**

VPI and MFV values were 0.42 ± 0.92 and 17.27 ± 5.34 cm/second, respectively, in the control group and 0.25 ± 0.50 and 12.82 ± 4.32 cm/second in patients with NAFLD (P< 0.01). The frequency of abnormal hepatic vein doppler waveform patterns (biphasic or monophasic) was significantly higher in patients with NAFLD (55.2%) versus control subjects (3.2%) (P < 0.001). There was no correlation between the degree of fat infiltration and VPI (P = 0.714), MFV (P = 0.911), or hepatic vein waveform pattern (P = 0.197). We found no correlation between liver enzyme levels and MFV or VPI. However, the rate of abnormal hepatic vein was higher in patients with enzyme levels that exceeded twice the normal value (P = 0.05).

**Conclusions:**

Patients with NAFLD have a high rate of abnormal hepatic vein doppler waveform patterns, and decreased VPI and MFV are suggestive of reduced vascular compliance in the liver. Elevated liver enzymes levels do not influence VPI or MFV, but patients with abnormal enzymes have higher rates of abnormal hepatic vein doppler waveform patterns.

## 1. Background

Nonalcoholic fatty liver disease (NAFLD) is the most common cause of abnormal liver function tests in hepatology practices. NAFLD affects approximately 15% to 25% of the general population worldwide [1] and is characterized by lipid deposition within the hepatocytes with no history of excessive alcohol consumption [2]. NAFLD is associated with central obesity, type 2 diabetes, dyslipidemia, high blood pressure, and metabolic syndrome (MetS) [3]. NAFLD is a spectrum that ranges from pure fatty liver to nonalcoholic steatohepatitis (NASH). Although it had been considered to be benign, recent studies have shown that liver fibrosis, cirrhosis, and liver failure can develop in patients with NAFLD. In 15% to 50% of cases, fibrosis exists at the initial diagnosis [4]. Liver ultrasonography is a noninvasive modality that is widely used to diagnose NAFLD and has a sensitivity of 82% to 89% and specificity of 93% in detecting fatty liver infiltration [5]. It is based on increased liver parenchymal echogenicity. Sonography of fatty infiltration can vary, depending on the amount of fat and whether deposits are diffuse or focal. Diffuse steatosis can be:

• Mild (grade 1): Minimal diffuse increase in hepatic echogenicity; diaphragm and intrahepatic vessel borders are visualized normally.

• Moderate (grade 2): Moderate diffuse increase in hepatic echogenicity; slightly impaired visualization of intrahepatic vessels and diaphragm.

• Severe (grade 3): Marked increase in hepatic echogenicity and poor penetration of the posterior segment of the right diaphragm [6].

Duplex doppler ultrasonography has become an important diagnostic method in the noninvasive evaluation of hepatic vasculature and certain hepatic parenchymal diseases [7]. Reduced hepatic blood flow is observed in experimental animals with fatty deposition in the liver, and similar data are expected in humans [4]. Recent articles suggest that NAFLD can change the waveform pattern of hepatic veins [7][8][9] and portal vein doppler indices [10][11]. NAFLD can also decrease hepatic artery resistance index [12]. Hepatic vein pulsatility is also flattened in obese patients, correlating with the grade of hepatosteatosis. The body habitus itself does not have an independent effect on hepatic venous waveform [13].

## 2. Objectives 

In this study, we investigated the association of nonalcoholic fatty liver disease with the doppler waveform pattern of the hepatic veins and portal vein doppler indices. We also examined the relationship between the degree of fatty infiltration in the liver and the waveform pattern of hepatic veins, portal vein pulsatility index, and mean flow velocity. This assay can be helpful in diagnosing NAFLD and its effects on liver hemodynamics, evaluating the natural course of NAFLD, and monitoring treatment efficacy on follow-up.

## 3. Patients and Methods

This case control study was performed from September 2009 to June 2010 in patients who were referred to the Gastrointestinal and liver diseases research center (GLDRC) of Guilan university of medical science and a private GI clinic in Rasht (the capital city of Guilan province) with elevated liver enzymes and hyperechogenic liver by B-mode ultrasonography. Other causes of liver enzyme elevation, such as alcoholic hepatitis, autoimmune hepatitis, viral hepatitis, Wilson’s disease, α1 antitrypsin deficiency, drug-induced hepatitis, and hemochromatosis, were ruled out. Patients with heart failure were excluded. Eleven patients had undergone a liver biopsy due to prolonged NAFLD. The control group was selected from healthy volunteers (Razi hospital personnel) after an evaluation, with no evidence of fatty liver by ultrasonography and normal liver enzyme levels. Cases with heart failure were excluded from the control group. The study protocol was approved by the ethics committee of the Gastrointestinal and Liver Diseases Research Center of Guilan University of Medical Science, and written informed consents (per the Helsinki declaration) was obtained from each participant. The study population included 31 patients (25 men and 6 women; mean age 40.65 ± 8.90 years, range 24–59 years) who were referred to the Hepatology Clinic of Razi Hospital Rasht, Iran, and 31 subjects in the control group (24 men and 7 women; mean age 38.19 ±11.98 years, range 22–61 years). After 8 hours of fasting, all patients and control subjects were examined by B-mode and duplex doppler ultrasonography using a3.5 MHz convex array transducer (Sonix OP, Canada) in the supine position by the same expert radiologist. The degree of fatty infiltration was graded from 0 to 3 with regard to increasing degrees of hepatic echogenicity with poorer visualization of the intrahepatic vessels and diaphragm, indicating absent, mild, moderate, and severe hepatosteatosis [6].Evaluation of the hepatic vein was performed in the left decubitus position using a right lateral intercostal approach during deep inspiration. The sample volume was positioned in the right hepatic vein 3-5 cm away from the inferior vena cava to prevent any influence of waveform alterations of the inferior vena cava and was adjusted to cover at least two-thirds of the vessel diameter {%7). The spectral analysis was recorded for at least 2-3 cycles, and the doppler angle was set to less than 60 degrees. We chose the right hepatic vain to avoid the effects of cardiac motion on vein pulsatility [14]. The flow pattern of the right hepatic vein was classified as triphasic (which is a regular pattern), biphasic, and monophasic. The triphasic wave pattern consisted of 2 antegrade peaks, followed by a short reversed flow; the biphasic waveform was defined as the loss of reversed flow and decreased amplitude of oscillations. The monophasic waveform was defined as a continuous flat wave without periodicity [13]. The portal vein examination was performed in the left decubitus position with the arms raised above the head. The sample volume was positioned in the main portal vein, proximal to the bifurcation to cover at least two-thirds of the vessel diameter, and the doppler angle was set to less than 60 degrees [10]. The peak maximum velocity (V max), peak minimum velocity (V min), mean flow velocity (MFV), and portal vein diameter were measured, and the vein pulsatility index (VPI) VPI = Vmax – Vmin/ Vmax [15]. A pathologist without any knowledge of the patient’s data evaluated the histological findings, and the nonalcoholic fatty liver disease activity score (NAS)[16] and stage were reported. The scoring system of NASH, developed by Brunt and colleagues, has been widely accepted. It unifies the lesions of steatosis and necroinflammation into a grade and those of fibrosis into a stage. The score is defined as the sum of the scores for steatosis (0-3), lobular inflammation (0-3), and ballooning (0-2), thus ranging from 0 to 8; fibrosis stage ranges from 0 to 4. NASH was defined as an NAS of ≥ 5, “borderline NASH” as NAS of 3 or 4, and “not diagnostic of NASH” as NAS of < 3 [16]. The fatty liver grading scale is based on ultrasonography The patients were divided to 2 subgroups, based on the liver enzyme levels: those with ALT levels less than twice the normal value (normal range = 35 IU/L) and more than twice the normal value [17]. Then, the doppler parameters were determined for each group. The statistical analysis was performed using SPSS for Windows, version 16.0. The distribution of data was analyzed by one-sample Kolmogorov-Smirnov test. Continuous variables were expressed as mean ± SD and as a percentage (%) for categorical variables. Fisher’s exact test was used as appropriate to analyze categorical variables, and Student’s t-test was used to analyze continuous variables. Fisher’s exact test was used to demonstrate the relationship between fatty liver grade and the presence of an abnormal hepatic vein Doppler waveform; one-way ANOVA was used to analyze the relationship between fatty liver grade and quantitative variables. All tests of significance were two-tailed, and P values that were less than 0.05 were considered statistically significant.

## 4.Results

The fatty liver was grade 1 in 9 patients (29%), grade 2 in 16 patients (52%), and grade 3 in 6 patients (19%). Mean BMI (body mass index) in the patients (29.23 ± 3.35 Kg/M2) was significantly higher than in the control group (23.79 ± 4.31 Kg/M2) (P < 0.001). There was no significant difference in mean portal vein diameter between patients (10.77 ±1.51 mm) and controls (10.35 ± 1.57 mm) (P = 0.379). The doppler flow pattern in the hepatic vein was triphasic in 17 (54.8%), biphasic in 10 (32.3%), and monophasic in 4 (12.9%) subjects with NAFLD and triphasic in 30 (96.8%) and biphasic in 1 (3.2%) patient in the control group; none had a monophasic wave pattern. The frequency of abnormal hepatic vein waveform (biphasic or monophasic) was significantly higher in patients with NAFLD than in controls (P < 0.001, OR = 23.33). However, we found no correlation between the degree of fat infiltration and abnormal hepatic vein waveform pattern (P = 0.197) Table 1. The MFV was 12.82 ± 4.32 cm/second in patients and 17.27 ± 5.34 cm/second in the control group (P < 0.01), Table 2. There was no significant difference in mean MFV between fatty liver grades (P = 0.911) Table 3. VPI was 0.25 ± 0.50 in patients and 0.42 ± 0.92 in control subjects (P < 0.001) (Table 2). There was no significant difference in mean VPI between fatty liver grades (P = 0.714) (Table 3).Liver enzyme levels were more than twice the normal value in 13 patients (41.9%). VPI and MFV values were 0.27 ± 0.54 and 12.18 ± 4.32, respectively, in the minor enzyme elevation group and 0.24 ± 0.43 and 13.60 ± 4.34, respectively, in patients with liver enzyme levels over twice the normal value. (P = 0.734 for VPI and P = 0.970 for MFV). The doppler flow pattern in the hepatic vein was abnormal (biphasic or monophasic) in 5 patients (27.8%) in the minor enzyme elevation group and 9 patients (69.2%) in the other group. The rate of abnormal hepatic vein waveform (biphasic or monophasic) was significantly higher in patients with enzyme levels more than twice the normal value than in those with levels less than twice the normal value (P = 0.05, OR = 5.85). In 11 patients who underwent liver biopsy, the nonalcoholic fatty liver disease activity score was 0-2 in 3 patients, 3-4 in 3 patients, and 5-8 in 5 patients. The pathological fibrosis stage was 0 in 8 and 1 in 3 patients. In patients with biopsy-proven fatty liver, the mean portal vein diameter was 10.59 ± 1.24 mm, VPI was 0.28 ± 0.06, and MFV was 13.89 ± 5.10. The doppler flow pattern in the hepatic vein was triphasic in 7 (64%) and biphasic in 4 (36%) patients, and none had a monophasic pattern.

**Table 1:** Distribution of the Right Hepatic Vein Flow Pattern According to Ultrasonographic Grading of the Fatty Infiltration of the Liver a

	**Grade I, No. (%) (n = 9)**	**Grade II, No. (%) (n = 16)**	**Grade III, No. (%) (n = 6)**
Triphasic	7 (77.8)	8 (50)	2 (33.3)
Biphasic	1 (11.1)	7 (43.8)	2 (33.3)
Monophasic	1 (11.1)	1 (6.2)	2 (33.3)

^a^ There is no correlation between the degree of fat infiltration and abnormal hepatic vein waveform pattern (biphasic or monophasic) (P = 0.197)

**Table 2:** The Portal Vein Pulsatility Index (VPI) and Mean Flow Velocity (MFV) in Patients with NAFLD and Control Group a

	**Control (n = 31)**	**NAFLD (n = 31)**	**Pvalue**
MFV b (cm/sec)	5.34 ± 17.27	12.82 ± 4.32	< 0.01
VPI b	0.92 ± 0.42	0.50 ± 0.25	< 0.001

^a^ VPI and MFV are significantly lower in patients with NAFLD than control group.

^b^ Abbreviations: MFV, Mean Flow Velocity; VPI, Vein Pulsatility Index

**Table 3:** The Portal Vein Pulsatility Index (VPI) and Mean Flow Velocity (MFV) According to Ultrasonographic Grading of the Fatty Infiltration of the Liver and Control Group a

	**Grade I (n = 9)**	**Grade II (n = 16)**	**Grade III (n = 6)**	**Pvalue**
MFV b (cm/sec)	13.29 ± 5.01	12.77 ± 4.17	12.28 ± 4.30	0.911
VPI b	0.26 ± 0.28	0.25 ± 0.64	0.24 ± 0.11	0.714

^a^ No significant correlation was found between the grade of fatty liver and VPI and MFV

^b^ Abbreviations: MFV, Mean Flow Velocity; VPI, Vein Pulsatility Index

## 5. Discussion

There was no significant difference in gender (P = 0.755) or age (P = 0.364) between case and control groups; thus, they did not influence the study results. The hepatic veins characteristically have a triphasic wave form, which reflects right atrial and inferior vena caval pressures. The pressure of the right atrium, respiration, and the compliance of the hepatic parenchyma influence the spectral wave of hepatic veins; thus, cardiovascular and liver parenchymal diseases, such as cirrhosis, liver transplant rejection, and Budd Chiari syndrome, can alter triphasic waveform patterns. Nontriphasic patterns are detected in normal populations as well [7]. Oguzkurt et al. reported that patients with fatty liver had a high rate of abnormal hepatic vein doppler waveform patterns, which can be biphasic or monophasic, but they found no correlation between the degree of fat infiltration and the hepatic vein waveform pattern [7]. Herbay et al. and Uzun et al. verified the effect of fatty infiltration on hepatic vein wave patterns. In a study by Uzun et al. sonographic grade of fatty infiltration of the liver correlated inversely with phasicity of hepatic venous flow [8][9]. In this study, we noted dampening of the hepatic vein spectrum (biphasic or monophasic) in patients with NAFLD, which might be due to hypertrophied hepatocytes that cause hepatic vein compression and subsequently decrease vein phasicity. We found no relation between the severity of fat deposition and abnormal hepatic vein wave patterns. Portal venous flow is classically described as being continuous in healthy subjects. Feeble pulsatility in rhythm with the cardiac cycle, however, has been noted in normal subjects. Marked pulsatility in the portal vein flow has been interpreted as a sign of congestive heart failure. Marked modulation of portal blood flow has been explained by exaggerated phasic pressure changes, transmitted from the right atrium [15].

In a report by Balci et al. venous pulsatility index (VPI) and mean flow velocity (MFV) of the portal vein were significantly lower in patients with diffuse fatty liver infiltration; these indices decreased as the severity of fatty infiltration increased [11]. Erdogmus et al. supported these findings in a similar study [10]. In our study, VPI and MFV were significantly lower in patients with NAFLD than in the control group, which could have been caused by impaired vascular compliance due to fat deposition in hepatocytes. There was no correlation between sonographic grading of fatty livers and MFV and VPI values. In all of these investigations [10][11] and our study, fatty liver infiltration was graded based on the ultrasonographic appearance changes, except for Oguzkurt et al. [7], who used an enhanced CT scan.However, ultrasonography has high sensitivity and specificity in detecting fatty liver infiltration [5]; the grading of fatty liver is subjective and can lead to interobserver disagreement. In addition, ultrasonography is unable to assess fibrosis, necrosis, and inflammation, which could influence MFV, VPI, and hepatic vein flow patterns, requiring liver biopsy to examine these conditions. Therefore, the differences in the effect of the severity of fatty liver on MFV, VPI, and hepatic vein spectrum might be due to these limitations. The number of patients could be another limitation (only 6Grade III patients in this study), and another study with more cases may be required. We found no correlation between liver enzyme levels and MFV and VPI. However, the rate of abnormal hepatic vein was higher in patients with enzyme levels more than twice the normal value. Because the number of biopsy-proven patients was not adequate for statistical analysis, another study on the pathology results should be performed with more participants.Patients with diffuse fatty liver infiltration have a higher rate of abnormal hepatic vein doppler waveform patterns (biphasic or monophasic) than the normal population, and VPI and MFV values are significantly lower than in healthy subjects. These findings suggest reduced vascular compliance in the liver in NAFLD. We found no significant correlation between sonographic fatty liver grade and MFV, VPI, or the rate of abnormal hepatic vein waveform. Elevated liver enzyme levels do not influence VPI and MFV, but patients with abnormal enzymes have higher rates of abnormal hepatic vein doppler waveform patterns.
